# Probing the Phase Composition and Surface Roughness in the Biological Response of Additively Manufactured Titanium Alloy Bioimplants

**DOI:** 10.1021/acsomega.5c08853

**Published:** 2025-12-23

**Authors:** Lu Yang, Yanhao Hou, Duo Meng, Axieh Bagasol, Fan Wu, David J. Browne, Denis Dowling, Weiguang Wang, Wajira Mirihanage

**Affiliations:** † Department of Materials, 5292 The University of Manchester, Manchester M13 9PL, U.K.; ‡ Department of Mechanical Engineering, School of Engineering, 7423 University Southampton, Southampton SO17 IBJ, U.K.; § Department of Mechanical and Aerospace Engineering, 5292 The University of Manchester, Manchester M13 9PL, U.K.; ∥ School of Mechanical & Materials Engineering, 8797 University College Dublin, Belfield, Dublin Dublin4, Ireland

## Abstract

Titanium alloys, mainly Ti-6Al-4V, are renowned for their impressive strength-to-weight ratio and stand as some of the most widely used metallic materials for bioimplants. Additive manufacturing introduces a paradigm shift in the short turnaround times for the availability of such implants. The biological performance of these implants is critical to ensure their success and is understood to be affected by a variety of factors, including surface characteristics and phase composition of the material, often determined by the manufacturing approach. The experimental investigation of the difference in biological performance caused by surface roughness and phase compositions resulting from manufacturing methods that involve laser powder bed fusion (LPBF) and hot isostatic pressing (HIP) has been conducted. Surface roughness was found to be the prevailing effect over the reported phase composition difference, with a relatively rougher surface seeming to be better for biological performance in this contribution. Meanwhile, HIP-ed Ti-6Al-4V samples exhibit better cell viability compared to that of the as-built LPBF-ed Ti-6Al-4V samples.

## Graphical abstract

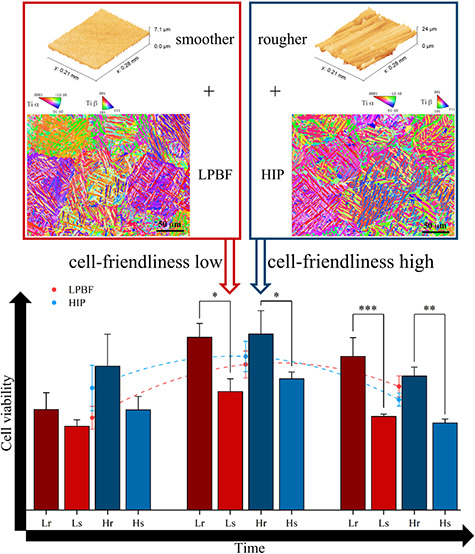

## 1 Introduction

The need for bone replacement and bioimplants is increasing due to the rising aging population, overweight, traffic accidents, and sudden shocks in sports. Bone implants are devices that work for bone replacement or bone regeneration and can reinforce the functions of damaged native bone tissue. Bone implants are made from a wide range of materials, including polymers, metals, ceramics, and composite materials.^1^ It is crucial to choose suitable materials for bone implants, as the material determines the function and lifespan of bioimplants for bone replacement.^2^ For metals, magnesium alloys, stainless steel, titanium alloys, and cobalt alloys are usually considered applicable. Among these, titanium alloys, such as commercially pure titanium for dental implants and Ti-6Al-4V for orthopedics, are some of the most commonly used alloys. As a bioimplant, Ti-6Al-4V (wt %), which contains crystallographic α and β phases, is highlighted because of its relatively good biocompatibility, as well as its favorable combination of lightweight and high strength.^3,4^

Additive manufacturing is the technique that fabricates products layer by layer according to the designed structure. Compared with traditional metallic manufacturing processes (e.g., casting and welding), additive manufacturing presents faster processes and higher material usage efficiency, while the production capacity is limited and equipment costs are high.^5^ Laser powder bed fusion (LPBF), also known as selective laser melting, is one of the additive manufacturing techniques that can make products with more efficient material powder utilization and a preferred shape, which is ideal for specific bone replacement.^6^ It is widely acknowledged that the microstructure of parts manufactured via additive manufacturing and subsequent heat treatments is different from that of traditional casting, forging, or wrought.^7^ Additionally, relatively enhanced cellular responses of LPBF-ed Ti-6Al-4V compared to electron beam melting have been reported by several researchers. The marginal difference could be attributed to differences in microstructure, physical, and chemical properties,^8,9^ suggesting the influence of metallic microstructure variations on biological performance, such as biocompatibility.

Hot isostatic pressing (HIP), achieved by applying inert gas pressure of around 100–200 MPa at a temperature of approximately 900 °C, is one of the heat treatments or postprocessing approaches in metallurgy.^10^ Generally, the LPBF-ed Ti-6Al-4V microstructure is dominated by fine martensitic α’ phase, and subsequent HIP modifies the LPBF-ed Ti-6Al-4V microstructure to a relatively coarse α phase with slightly more β phase.^11^ Therefore, in this contribution, HIP is used to compare the possible effect of its consequently different microstructure and chemical distribution on the biological performance of the Ti-6Al-4V bioimplant. In contrast, the martensitic phase produced by LPBF can be beneficial for biological performance. It was pointed out that the water-quenched Ti-6Al-4V microstructure, which is the combination of the maximum amount of martensite phase and the minimum amount of β phase, can be relatively more suitable for bioimplant applications owing to better cell viability compared to the microstructure with the majority of α or β phase.^12^ However, they reported that the martensitic microstructure can exhibit inferior cell proliferation. Therefore, to further analyze the biological performance, it is necessary to consider the microstructure difference between LPBF-ed and HIP-ed Ti-6Al-4V.

The biological performance of titanium bioimplants relies on various factors, including topology, mechanical stiffness, chemical composition, and surface properties. Surface property is one of the most significant factors influencing performance under both *in vitro* and *in vivo* environments,^13^ including surface energy, surface roughness, surface wettability, surface topology, porosity, etc.^14–16^ Among these, surface roughness is particularly important and will be one of the focuses of this research. Considerable research has been conducted on the effect of the surface roughness of titanium alloys on their biological performance. However, it is difficult to define a specific optimal surface roughness value. In general, multiple studies have reported that acceptable biological performance can be achieved with surface roughness (Sa value) ranging from around 1 to 15 μm. For example, Straumal et al. reported that the best cell adhesion was achieved by grinding titanium implants to a surface roughness (Sa) of 0.079 μm,^17^ while Yu et al. proved that a surface roughness (Sa) of approximately 14 μm can facilitate better cell adhesion and proliferation on additively manufactured titanium implants.^18^ Studies by Wennerberg and Albrektsson investigated titanium implants with a series of surface roughness levels (smoother surfaces Sa < 0.5 μm; moderate roughness Sa 1–2 μm and rougher surfaces Sa > 2 μm) and obtained optimal osteointegration within the moderate range.^19,20^ With all of the observations above, it is critical to perform a systematic study in order to know the optimal surface roughness range.

In addition to surface roughness, the biological performance of titanium bioimplants also depends on ion release, which is related to the metallic corrosion process, chemical distribution, and texture of the metallic bioimplants. It is noteworthy that the Ti-6Al-4V bioimplant used nowadays is recognized as biocompatible, as its ion release levels, which should be minimized as much as possible, are below the acceptable limit for daily human intake.^12,21,22^ In Ti-6Al-4V, the major ion release potentials are related to Al ions and V ions, which are considered to be toxic. The most release of Ti and V ions was observed between weeks 4 and 12 after implementation.^22^ Furthermore, most work concluded that the crystallographic β phase, which has a higher content of the toxic V element in Ti-6Al-4V, can be detrimental to the biocompatibility of titanium bioimplants.^9^ The release of the V element after long-term immersion in body fluid can limit biocompatibility (e.g., cell proliferation and differentiation) and cause neuroinflammation and Alzheimer’s disease.^23–25^ Thus, comparison of the biological performance of different phases and chemical distribution, by examining the extent or distribution of toxicity, can extend the understanding of the fabrication of advanced metallic biomaterials.

Although extensive research has been conducted on the relationship between the biological performance of titanium bioimplants and their surface properties or corrosion resistance,^26,27^ the connection between microstructural characteristics and the biological performance of titanium bioimplants has not been fully explored. Furthermore, although significant progress has been made in understanding how surface properties, particularly surface roughness, affect the biological performance of Ti-6Al-4V, a systematic and precise analysis of the surface roughness is still required. Therefore, this study provides a detailed analysis and comparison of how different surface roughnesses, along with the microstructure of LPBF-ed and HIP-ed Ti-6Al-4V, impact biological performance, particularly in terms of cell viability.

## 2 Methodology

### 2.1 Material Fabrication and Preparation

In this research, grade 23 Ti-6Al-4V alloy extralow interstitial powders (Stryker, Ireland) of ASTM B348–19, with powder sizes ranging from 21 to 48 μm, were deposited using the Renishaw 500S AM system after preheating the build plate and maintaining its temperature at 170 °C throughout the process. The utilized laser power, point distance, hatch spacing, exposure time, and layer thickness were, respectively, 400 W, 80 μm, 100 μm, 60 μs, and 60 μm. Afterward, half of the samples (8 discs out of a total of 16) were heat-treated using HIP at 920 °C and 100 MPa for 2 h in an inert gas atmosphere.^7^ Finally, the samples of each manufacturing method were extracted from the whole build volume and sliced into circular discs with a 6 mm diameter and 2 mm thickness by electrical discharge machining (EDM). Notably, the as-built LPBF-ed and HIP-ed samples, after slicing with EDM, are not suitable for direct characterization; thus, further grinding and polishing were conducted. Details will be illustrated in the following methodology of characterization.

### 2.2 Material Characterization

#### 2.2.1 Microstructure and Chemical Distribution

Samples were ground using silicon carbide abrasive papers with P120, P240, P400, P800, P1200, and P2400 grit sizes. After further polishing with a 0.04 μm colloidal silica solution to mirror surface finish, microstructures of LPBF-ed and HIP-ed Ti-6Al-4V were revealed by Electron Backscattered Diffraction (EBSD) in the TESCAN Mira3 large-chamber Scanning Electron Microscope (SEM), with 20 kV accelerating voltage, 27 mm working distance, and 0.3 μm step size. The phase maps were generated by the MTEX toolbox in MATLAB from the EBSD CPR data files. In addition, the chemical distributions of LPBF-ed and HIP-ed Ti-6Al-4V were also investigated by Energy Dispersive X-ray Spectroscopy (EDS) in the TESCAN Mira3 small chamber SEM with a 20 kV accelerating voltage and a 15 mm working distance. The size of each map is 285 μm × 215 μm.

#### 2.2.2 Surface Roughness

The surface roughness of the samples was characterized with the Keyence X200 K Confocal Laser Scanning Microscope (CLSM) to ensure that the accurate surface roughness was correctly created by grinding with silicon carbide abrasive papers. Data analysis and surface roughness were conducted and displayed using Gwyddion software (Czech Metrology Institute, Czech). Notably, two ranges of surface roughness were prepared for each manufacturing group, with four groups obtained eventually. The surface roughness levels were carefully selected within the range of Sa that is suitable for cell attachment and *in vitro* biological evaluation. Table 1 lists the sample group details based on the manufacturing methods and surface roughness ranges, with the details for each sample found in Figures S1 and S2. *L*_*r*_ represents LPBF-ed samples with a rougher surface (average Sa = 1.38 μm), *L_s_* represents LPBF-ed samples with a smoother surface (average Sa = 0.32 μm), *H_r_* represents HIP-ed samples with a rougher surface (average Sa = 1.25 μm), and *H_s_* represents HIP-ed samples with a smoother surface (average Sa = 0.48 μm). This definition of smooth and rough ranges was based on the values reported as typical optimal levels for cellular response, as detailed in the introduction. Furthermore, these values also balance the requirement to employ two adequately distinct ranges of surface roughness levels for the analysis.

**Table 1:** Surface Roughness for LPBF-Ed and HIP-Ed Ti-6Al-4V Samples Grouped for Controlling Variables According to the Rougher and Smoother Surfaces

Group	*L*_*r*_	*L*_*s*_	*H*_*r*_	*H*_*s*_
Manufacturing methods	LPBF		HIP post heat treatment	
Averaged roughness Sa (μm)	1.38 ± 0.89	0.32 ± 0.09	1.25 ± 0.62	0.48 ± 0.35

### 2.3 Biological Characterization

#### 2.3.1 Cell Culture and Seeding

Human adipose-derived stem cells (hADSCs, passages 6–8, StemPro, Invitrogen, USA) were subcultured in MesenPRO RS basal medium (Invitrogen, USA) in T75 flasks (Sigma-Aldrich, UK) under standard conditions (37 °C, 5% CO_2_, and 95% humidity). Cells were harvested when they reached approximately 80% confluence using 0.05% trypsin (Invitrogen, USA). All samples were sterilized using ethanol and phosphate-buffered saline (PBS, Sigma-Aldrich, UK) and air-dried before cell seeding. Approximately 40000 cells suspended in 0.2 mL of medium were seeded onto each sample in a 48-well plate and then cultured in the incubator under standard conditions, with the culture medium refreshed every 2 days.

#### 2.3.2 Cell Proliferation Analysis

The viability of hADSCs was assessed via the Alamar Blue assay after 1, 3, and 5 days of cell seeding. On each day, all samples were transferred to a new well plate, and 0.2 mL of medium containing 0.001% resazurin sodium salt (Invitrogen, USA) was added to each sample. After incubating for 4 h under standard conditions in the dark, 150 μL of medium was collected, and the fluorescence intensity was measured at excitation/emission wavelengths of 540/590 nm using a TECAN Infinite 200 plate reader (Tecan, Switzerland).

#### 2.3.3 Bioimaging

Confocal microscopy imaging was conducted to further assess the status of cell adhesion, spreading, proliferation, and differentiation, considering the fixed-cell-seeded samples after 5 days of proliferation. In brief, cell-containing samples were fixed in a 10% formalin solution (Sigma-Aldrich, UK) for 40 min, followed by a rinse with PBS. The samples were then immersed in PBS containing 0.1% Triton X-100 (Sigma-Aldrich, UK) for 7 min, rinsed again with PBS, and incubated with PBS supplemented with 7% fetal bovine serum (FBS, Sigma-Aldrich, UK) for 30 min at room temperature. Subsequently, Alexa Fluor 594 Phalloidin (Invitrogen, USA) was applied at the manufacturer-recommended dilution (1:400) for 45 min in the dark to stain cellular actin, and cell nuclei were stained with 4’,6-diamidino-2-phenylindole dihydrochloride (DAPI, Invitrogen, USA) at a 1:800 dilution. Confocal images were captured using a Leica SP8 LIGHTNING confocal microscope (Leica, Germany).

### 2.4 Data Analysis

All experiments were conducted at least three times, and the results were reported as the mean value and standard deviation. Data analysis was conducted using OriginLab (OriginLab Corporation, USA) by employing one-way analysis of variance (ANOVA) followed by Tukey’s post hoc test. Statistical significance levels were set as * *p* < 0.05, ** *p* < 0.01, and *** *p* < 0.001.

## 3 Results

### 3.1 Microstructure, Phase, and Elemental Distribution

Microstructure characterization results are displayed in Figure 1 for representative EBSD images and phase distribution, as well as Figure S3 for the elemental distribution of Ti-6Al-4V room-temperature microstructures (in Figure 1a,c) manufactured by LPBF and HIP. It can be observed that the microstructures of Ti-6Al-4V manufactured by both LPBF and HIP are featured by basket-weave laths. Furthermore, in Figure 1, the microstructure of LPBF-ed Ti-6Al-4V is mainly martensitic α’ phase with a smaller or finer grain size (mean value of 2.71 μm) and a tiny amount of β phase (0.74%). The microstructure of HIP-ed Ti-6Al-4V is dominated by the α phase with a relatively higher β phase (1.23%) and larger α grain size (mean value of 3.05 μm). This is consistent with the results in other research.^7^ Furthermore, a more homogeneous β phase distribution in HIP-ed samples is observed in Figure 1d, while the β phase distribution in LPBF-ed samples in Figure 1b is more concentrated in the bottom-left region.

**Figure 1:**
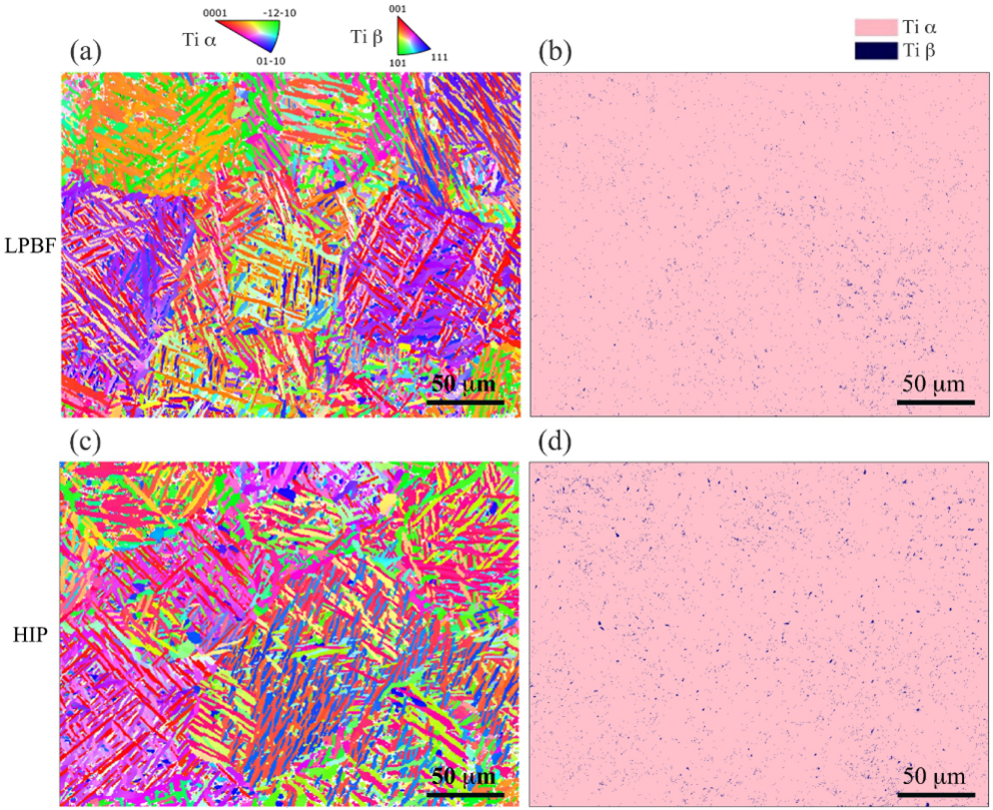
EBSD images and phase maps of Ti-6Al-4V samples: (a) EBSD of a representative LPBF-ed sample; (b) phase map of a representative LPBF-ed sample; (c) EBSD of a representative HIP-ed sample; and (d) phase map of a representative HIP-ed sample.

As mentioned in the Introduction, surface chemistry can be crucial for the biological performance of bioimplants, as ion release may have toxicity issues^25^ (Figure S3 displays the elemental distribution for LPBF-ed and HIP-ed samples). It is worthwhile to mention that the microstructure and phase map, as well as elemental distribution demonstrated in Figures 1 and S3 a are typical and representative of all LPBF-ed samples and HIP-ed samples used in this work, as the microstructure features and phase distribution of samples extracted in the same way and produced by each manufacturing method are similar across the whole build volume. This has been well illustrated in previously reported works.^28,29^

### 3.2 Surface Roughness

As mentioned in the Introduction, surface roughness is one of the most significant factors for the biological performance of bioimplants. Therefore, to enlarge the difference caused by surface roughness, two different ranges of surface roughness are aimed to be analyzed. These smoother surfaces (Sa around 0.40 μm) and rougher surfaces (Sa around 1.30 μm) were chosen according to works^17–19,30–32^ mentioned in the introduction. Nevertheless, it is still difficult to ensure that the surface roughness is exactly the same across all the samples by mechanical hand grinding, even though the surface roughness was carefully created and then characterized with the CLSM. Using the silicon carbide paper abrasive grits investigated, the maximum roughness (Sa) achieved by mechanical grinding is approximately 2.30 μm, which can also vary at different positions on the sample surface. The measured average surface roughness is representatively displayed in Figure 2 and listed in detail in Table 1.

**Figure 2:**
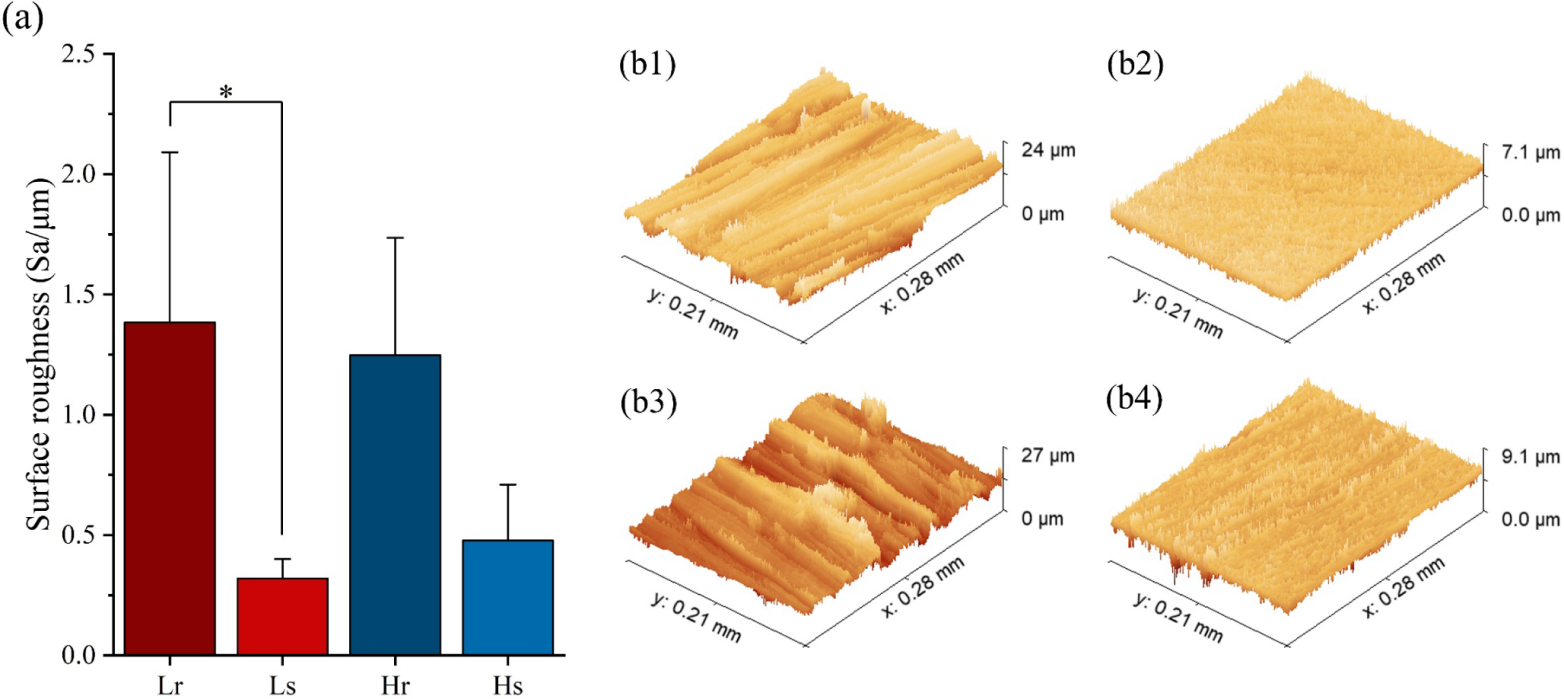
Surface roughness (Sa) of Ti-6Al-4V samples. (a) Average surface roughness for four sample groups; (b1) surface morphology of a representative *L_r_* sample; (b2) surface morphology of a representative *L_s_* sample; (b3) surface morphology of a representative *H_r_* sample; and (b4) surface morphology of a representative *H_s_* sample.

### 3.3 Biological Testing

Figure 3 shows the fluorescence intensities of LPBF-ed and HIP-ed samples with different surface roughnesses from days 1 to 5, which are proportional to the metabolic activities of the living cells, reflecting the cell proliferation status. Results show that the fluorescence intensities of the *L_r_* and *H_r_* groups were significantly higher than those of the *L_s_* and *H_s_* groups at all time points. Meanwhile, the fluorescence intensities of LPBF-ed samples were generally lower than those of HIP groups, particularly on days 1 and 3, but without statistical differences. This was probably due to the two groups reaching overconfluency at different times. In simpler terms, cells fully cover HIP-ed samples earlier than LPBF-ed samples (see dashed lines). Confocal microscope images in Figure 3 show that the surfaces of both group samples were fully covered by cells, confirming the overconfluency.

**Figure 3:**
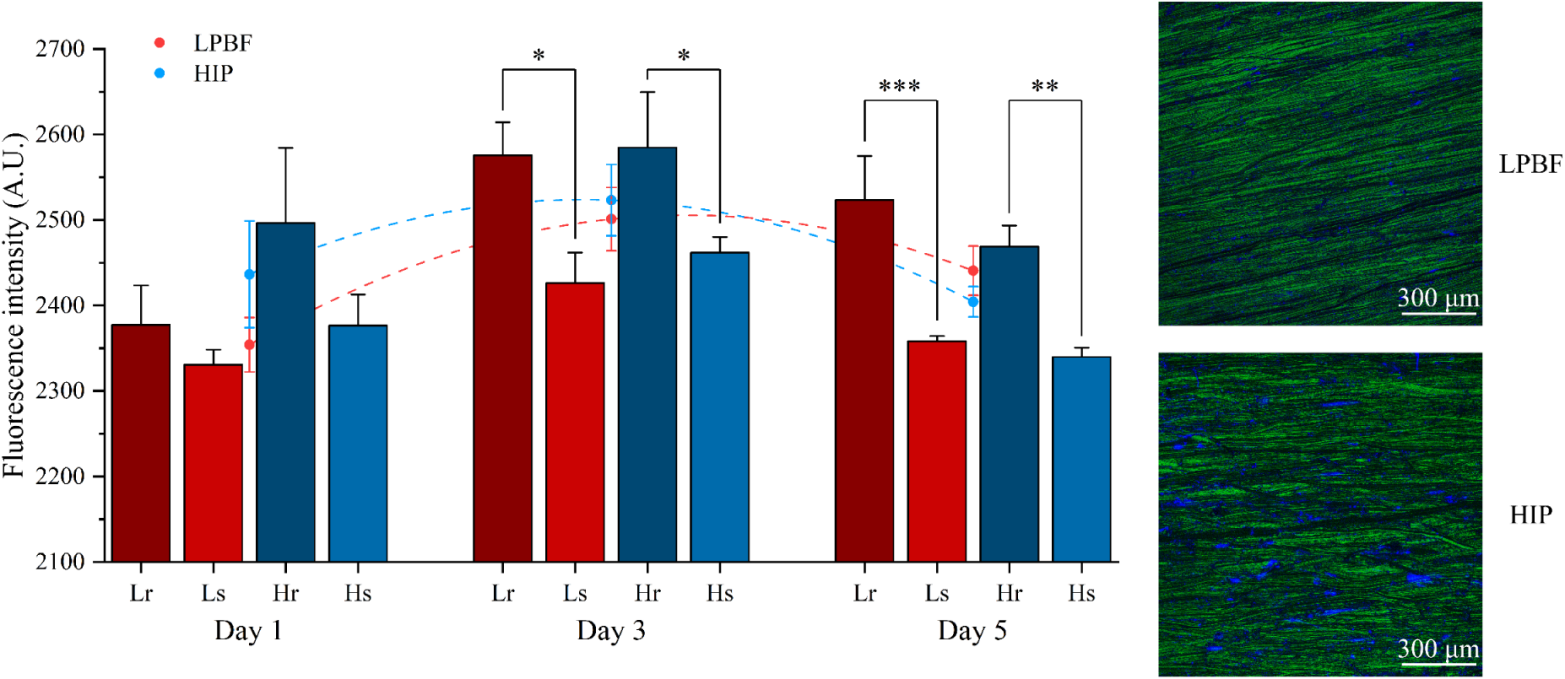
HADSC viability and proliferation results on LPBF-ed and HIP-ed Ti-6Al-4V samples with different surface roughness. Red and blue dots represent the mean fluorescence intensity of LPBF (*L_r_* and *L_s_*) and HIP (*H_r_* and *H_s_*) groups on each day, and the dashed lines represent the curve-fitted cell proliferation trends. Alexa Fluor 488 (green) was used for F-actin, and 4′,6-diamidino-2-phenylindole (DAPI) (blue) for nuclei.

## 4 Discussion

When producing a component from a given material, using different manufacturing routes can produce an exact similar shape. But the properties and functionality can significantly vary due to the internal differences that occur as a result of processing history.^33,34^ These internal differences can include microstructures, phase compositions, and chemical distributions, which are potential factors for the biological performance of bioimplants. However, only a few studies have been conducted on the microstructure and phase distribution for the biological properties of Ti-6Al-4V bioimplant.^35,36^ This contribution is an initial step to compare the effect that microstructure, phase, and chemical distribution produced by LPBF and HIP have on Ti-6Al-4V biological performance, with the effect caused by surface roughness. Both rougher and smoother surface roughness were created on Ti-6Al-4V samples produced by LPBF and postheat-treated by HIP. The LPBF-ed samples have a finer martensitic α’ microstructure and a more segregated chemical distribution. The HIP-ed samples contain an α microstructure with a larger grain size and a more homogeneous chemical distribution. To compare the effect of each variable, including surface roughness and microstructural features, samples were divided into four groups based on the method of control variables. To investigate the assumption and the effect that manufacturing methods (or sample types) have on the cell viability of Ti-6Al-4V, samples manufactured by different methods with a similar surface roughness range were selected using the control variable method. To be more specific, the *L_r_* and *H_r_* groups were compared to discover the effect that different manufacturing methods have on the Ti-6Al-4V samples with rougher surfaces. Meanwhile, the *L_s_* and *H_s_* groups were compared to discover the effect that different manufacturing methods have on the Ti-6Al-4V samples when the surface roughness is smoother. When processing conditions are considered, high laser power, inducing a high cooling rate, which is common in most LPBF processes, can produce Ti-6Al-4V with a very fine martensitic microstructure and much more limited β phases, where the toxic vanadium element segregates. In practical conditions, the optimal LPBF parameters are around the medium range when mechanical properties are concerned. However, in the case of biological performance, the optimal parameters are yet to be explored. Therefore, this study starts with processing parameters that produce relatively less β compared to the standard level of the β phase. When considering the metallurgical phase composition of Ti-6Al-4V, it was reported that the α phase is rich in Al, which is an α stabilizer and relatively inert in human body fluid, while the β phase contains more V, which is a β stabilizer and can be relatively toxic in the biological environment.^37,38^ As the amount of V element (4 wt %) that segregates in the β phase is the same for both LPBF-ed and HIP-ed Ti-6Al-4V, it can be deduced that the vanadium elemental distribution is more homogeneous in HIP-ed samples with more β phase, while vanadium distribution is more segregated in LPBF-ed samples with less β phase, according to our EDS measurements. Therefore, it is possible to propose that HIP-ed Ti-6Al-4V samples may have better biological properties, including cell viability, compared to those of LPBF-ed samples. In our case, EDS measurements provide a moderate resolution. However, we assume that the measurements provide a basic overview that is adequate for this analysis. This can be further understood through the higher mass diffusion opportunity in the HIPing processes carried out at higher temperatures for a considerable amount of time.

Surface roughness plays a crucial role in the biological performance of Ti-6Al-4V bioimplant, and the samples with different surface roughness were compared within the same manufacturing method. The selected surface roughness range (0.3–1.4 μm) was intentionally chosen to represent the moderate roughness level that has been widely reported as optimal for bioimplant surfaces supporting cell adhesion and early tissue integration. Surfaces that are too smooth (<0.1 μm) generally provide limited anchorage points and reduced protein adsorption, leading to weaker initial cell attachment, whereas excessively rough surfaces (>2 μm) can impair uniform cell spreading and may cause localized stress concentrations or bacterial colonization.^39–41^ Numerous studies on stem cells have shown that moderate nanometer to submicron roughness (∼0.2–1.5 μm) enhances adhesion, proliferation, and early osteogenic signaling compared to both smoother and rougher counterparts.^42–44^ Thus, the designed *L_r_* was compared with *L_s_* in terms of samples manufactured by LPBF, and *H_r_* was compared with *H_s_* in terms of samples further postheat treated by HIP. It can be observed from Figure 3 that rougher surfaces generally exhibit significantly higher cell viability compared to those with smoother surfaces throughout all time points. This can be attributed to the larger area for cell attachment and the production of related proteins and growth factors.^32,45^ The obtained result is aligned with several previous works, which reported good cell viability on surfaces with similar roughness ranges.^19,32^ Studies explained that smoother surface roughness can be detrimental to interlocking reactions at the interface region, while too-rough surfaces can cause some limitations, including ion release and peri-implantitis.^30^

Beyond surface roughness, elemental distribution may also affect the biological performance of the Ti-6Al-4V bioimplant. The results in Figure 3 show that, although there are no statistical differences, HIP-ed samples show higher fluorescence intensities than LPBF-ed samples on days 1 and 3 for both rougher and smoother groups, potentially demonstrating better cell viability. This is probably because the HIP process weakens the concentrated distribution of toxic vanadium in LPBF-ed Ti-6Al-4V. Besides, the slightly higher results of the LPBF group than the HIP group on both rougher and smoother samples on day 5 may be attributed to the overconfluence of cells on the sample, which can be observed from the fitted dashed lines. Before day 5, the attached cells constantly proliferated on all samples, and thus, the fluorescence intensity increased from day 1 to day 3. However, the attached cells in HIP groups reached full confluence (cells have covered the entire surface area of the sample with no space left for further growth) earlier than the LPBF groups, and thus, the fluorescence intensity started to drop earlier than the LPBF group, as the sample could not further sustain cell growth. Despite the overconfluence, the observation suggests that chemical distribution could be a potential factor in biological performance when comparing samples with similar roughness.

Additionally, corrosion and released ions from the bioimplant material could play a role. However, corrosion-related ion release typically occurs at a slow rate, potentially influencing long-term biological responses.^23,46,47^ The short duration of the Alamar Blue assay may not have been sufficient to capture its long-term effects, or the released ions in 5 days are too little to affect biological performance.

Overall, through all time points, it can be observed that the fluorescence intensity difference is more significant when comparing different surface roughness ranges, and the element-related fabrication process also affects the biological performance before the overconfluence of cells. Despite these factors, the results suggest that the difference in Alamar Blue assay results is more related to the different surface roughness of Ti-6Al-4V bioimplant and is less correlated to different microstructure, phase, or chemical distribution. In other words, it can be concluded that surface roughness has a more dominant impact on cell viability compared to microstructural differences or chemical distribution variations induced by different manufacturing methods.

## 5 Conclusions

The effects of different surface roughness and microstructure-driven chemical distributions appearing across Ti-6Al-4V bioimplant for cell viability and proliferation were experimentally examined and analyzed. The considered material chemical distributions directly resulted from the manufacturing sequences of Ti-6Al-4V. This contribution can be considered as one of the initial experimental works to analyze correlations between microstructural characteristics and the biological performance of metallic bioimplants, and the comparison of those with the surface characteristics. The surface roughness is found to affect the biological performance of additively manufactured Ti-6Al-4V bioimplant, and this influence is at a dominant position, stronger than other factors such as phase and chemical distribution (determined by the manufacturing method and consequent microstructure), despite the consideration of cytotoxicity from the vanadium element segregated in the β phase. While the manufacturing route and history significantly determine microstructural features, HIP postheat treatment can be advantageous for Ti-6Al-4V bioimplant than as-built LPBF additive manufacturing. However, further research is essential to investigate the extended and detailed relationships among microstructural features. The planned validations include high-accuracy chemical distribution analysis, such as nanoscale secondary ion mass spectrometry in relation to microscopic details of the related biological performances, long-term ion release studies through *in vitro* degradation tests, *in vitro* osteogenesis characterization through alkaline phosphatase assay and alizarin Red-S staining, and *in vivo* biological performance characterization considering a rat model with histomorphometry analysis.

## Supplementary Material



## Data Availability

The data underlying this study are available in the published article and its Supporting Information.

## Notes

Surface roughness profile and EDS maps for chemical distribution of all Ti-6Al-4V samples (PDF)
