# Supplementary material for: Probing the Phase Composition and Surface Roughness in the Biological Response of Additively Manufactured Titanium Alloy Bioimplants
Source: ACS Omega. 2025 Dec 23;11(1):1388–95. doi: 10.1021/acsomega.5c08853 (PMC12809321; doi:10.1021/acsomega.5c08853)
Supplement: Supplementary file 1 [file ao5c08853_si_001.pdf]

Probing phase composition and surface roughness in the biological response of additively manufactured titanium alloy bioimplants

Lu Yang [1, †], Yanhao Hou [2, †], Duo Meng [3], Axieh Bagasol [4], Fan Wu [1], David J. Browne [4], Denis Dowling [4], Weiguang Wang [2, *], Wajira Mirihanage [1, *]

[1] *Department of Materials, The University of Manchester, Manchester M13 9PL, UK*

[2] *Department of Mechanical Engineering, School of Engineering, University Southampton, Southampton SO17 IBJ, UK*

[3] *Department of Mechanical and Aerospace Engineering, The University of Manchester, Manchester M13 9PL, UK*

[4] *School of Mechanical & Materials Engineering, University College Dublin, Belfield, Dublin 4, Ireland*

* *Corresponding author email: weiguang.wang@soton.ac.uk; wajira.mirihanage@manchester.ac.uk*

† *These authors contributed equally to this work.*

Supporting Information

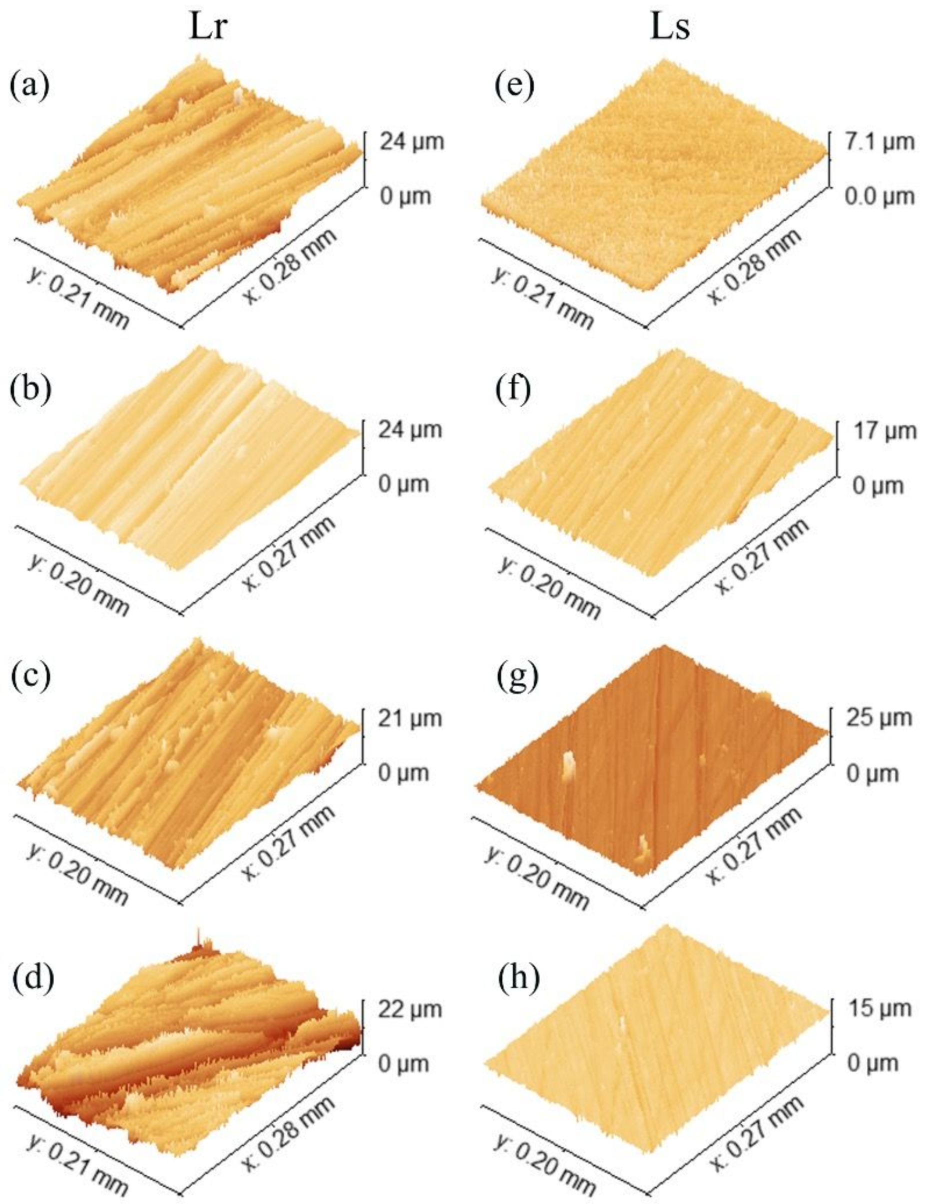

Figure S1. Surface roughness of each LPBF-ed sample. (a-d) L_r group samples, (e-h) L_s group samples.

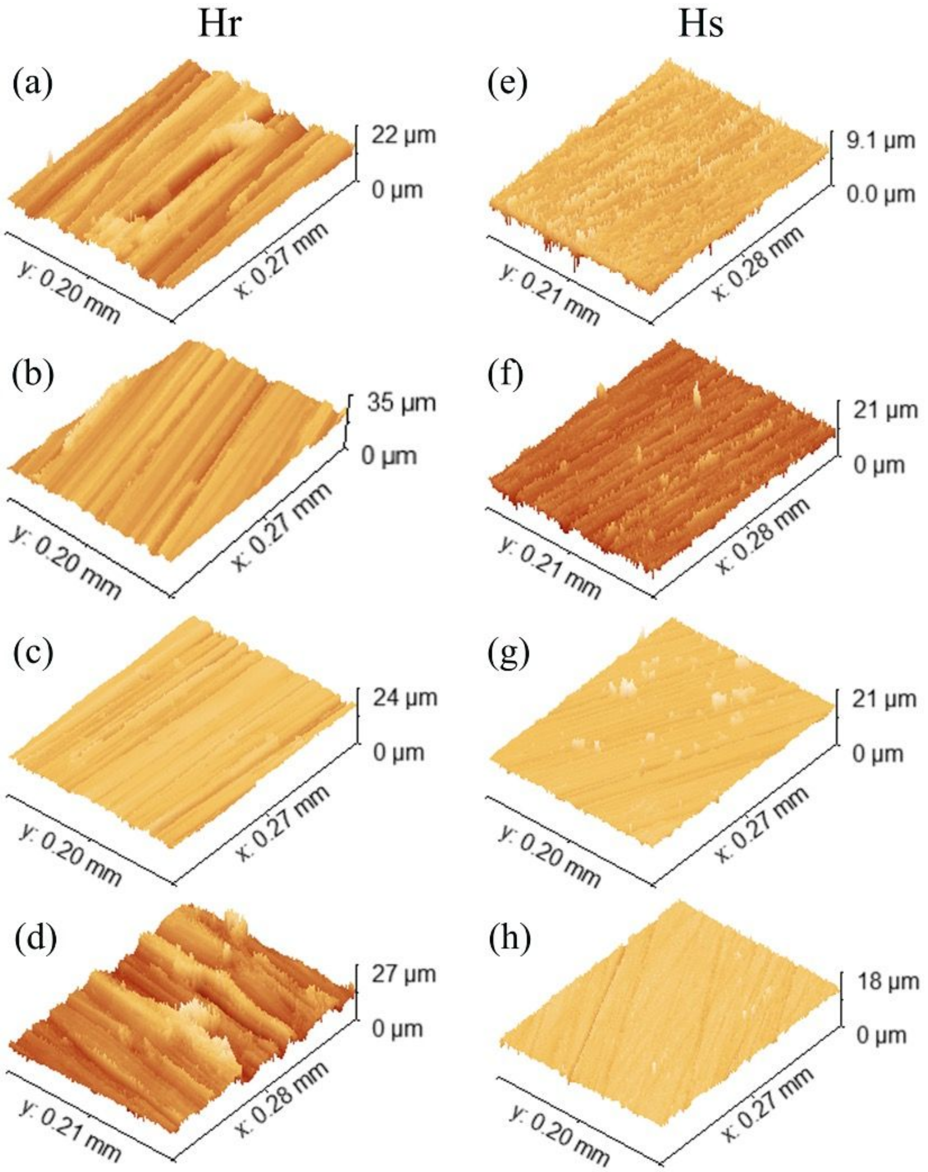

Figure S2. Surface roughness of each HIP-ed sample. (a-d) H_r group samples. (e-h) H_s group samples.

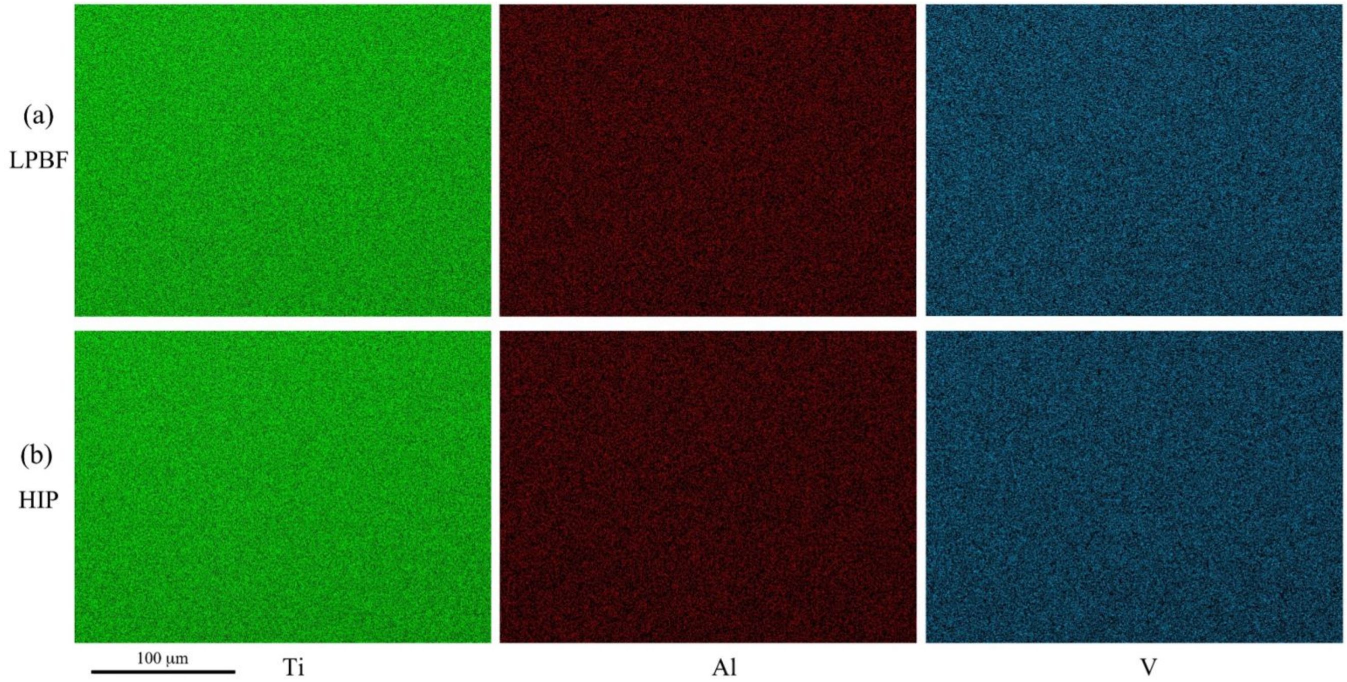

Figure S3. EDS maps for chemical distribution of Ti-6Al-4V samples. (a) Representative LPBF-ed sample, (b) Representative HIP-ed sample.
